# Cancer Stem Cells and Targeting Strategies

**DOI:** 10.3390/cells8080926

**Published:** 2019-08-18

**Authors:** Luisa Barbato, Marco Bocchetti, Anna Di Biase, Tarik Regad

**Affiliations:** 1 The John van Geest Cancer Research Centre, School of Science and Technology, Nottingham Trent University, Clifton Lane, Nottingham NG11 8NS, UK; 2 Department of Precision Medicine, University of Campania “Luigi Vanvitelli”, 80138 Naples, Italy

**Keywords:** cancer stem cells (CSCs), chemoresistance, chemotherapy, therapy, microenvironment, cancer

## Abstract

Chemoresistance is a major problem in cancer therapy as cancer cells develop mechanisms that counteract the effect of chemotherapeutic compounds, leading to relapse and the development of more aggressive cancers that contribute to poor prognosis and survival rates of treated patients. Cancer stem cells (CSCs) play a key role in this event. Apart from their slow proliferative property, CSCs have developed a range of cellular processes that involve drug efflux, drug enzymatic inactivation and other mechanisms. In addition, the microenvironment where CSCs evolve (CSC niche), effectively contributes to their role in cancer initiation, progression and chemoresistance. In the CSC niche, immune cells, mesenchymal stem cells (MSCs), endothelial cells and cancer associated fibroblasts (CAFs) contribute to the maintenance of CSC malignancy via the secretion of factors that promote cancer progression and resistance to chemotherapy. Due to these factors that hinder successful cancer therapies, CSCs are a subject of intense research that aims at better understanding of CSC behaviour and at developing efficient targeting therapies. In this review, we provide an overview of cancer stem cells, their role in cancer initiation, progression and chemoresistance, and discuss the progress that has been made in the development of CSC targeted therapies.

## 1. Introduction

In 2018, the World Health Organization (WHO) asserted that cancer is responsible for the death of 9.6 million people worldwide [1]. The data also suggest that despite the availability of different drugs, their efficacy requires further improvement with a focus on cancer complexity. In this intricate system, cancer stem cells play a crucial role, as they mediate chemoresistance and cancer recurrence. It is now clear that cancer stem cells (CSCs) represent a small population of cancer cells within a tumour and that they have typical characteristics related to stem cells. Like their stem cell counterparts, CSCs possess ‘stemness’ properties which are reflected in their capacity to self-renew and to generate differentiated cells that contribute to tumour heterogeneity. CSCs appear to be generated from mutations affecting adult stem cells that are at the source of organogenesis and tissue homeostasis [2,3,4]. Although debates about their origin persist, there is an urgent and continuous need to better understand the function of CSCs in cancer initiation and progression and, more importantly, develop CSC-specific targeting strategies.

Chemotherapy induces apoptosis by damaging DNA and/or inhibiting mitotic division. However, this therapeutic option is valid for highly dividing cancer cells and therefore has the potential to “miss out” slow dividing cells, leading to cancer relapse and recurrence in treated patients. Unfortunately, follow up chemotherapy that attempted to destroy refractory cancers cells has been mostly unsuccessful and was associated with a decrease in survival rates of treated cancer patients [5,6]. Due to their slow rate of division, CSCs mediate chemoresistance and recurrence following chemotherapy; however, additional mechanisms that confer CSCs a survival advantage include the expression of the ATP-binding cassette (ABC) transporters, a higher expression of aldehyde dehydrogenases (ALDHs), an increased resistance to apoptosis, and a timely activation of the DNA damage sensor and repair machinery [7]. Other mechanisms, that are not discussed in this review, involve epigenetic factors that play a role in regulating gene expression at the transcriptional or post-transcriptional levels [8,9]. All these properties and their clinical implications point to the importance of investigating and targeting CSCs.

It is well known that the microenvironment or niche is essential for stemness maintenance, and this also applies to CSCs where cell‒cell interactions within the niche are required for CSCs’ role in cancer initiation and progression [10]. The CSC niche is a complex environment that contains different types of cells, such as cancer-associated fibroblasts (CAFs), endothelial and immune cells, and mesenchymal stem cells (MSCs). These cells communicate through secreted growth factors and cytokines to promote tumorigenesis, angiogenesis, invasion and metastasis. The microenvironment of CSCs also contributes to CSCs’ resistance to drugs and other cancer therapies, which promotes cancer recurrence [11]. Several therapeutic strategies have been developed to target CSCs with the aim of preventing cancer recurrence. Although progress has been made through the development of targeting strategies, their therapeutic efficacy remains insufficient due to the absence of specific targetable biomarkers. In this review, we provide an overview of CSCs, their role in cancer initiation and progression, and discuss the targeting strategies that are being developed to target these highly aggressive cancer cells.

## 2. Cancer Stem Cells

“Omnis cellula e cellula” (“every cell stem from another cell”), with those words Rudolf Virchow, a German pathologist, gave the base of the cellular pathology. He had already postulated that the disease is due to changes happening in normal cells and that a tumour originates from immature cells [12,13]. However, the term cancer stem cell was coined by Sajiro Makino through the identification of a subpopulation of cells that were resistant to chemotherapy and with genetic characteristics that were different from the Bulk [13]. It was through studies carried out using the in vitro colony formation assay that Makino theory could be confirmed, and that suggested that a tumour could have originated from cells that had characteristics attributable to stem cells [14]. Further confirmation of Virchow’s idea came later from experiments using acute myeloid leukaemia (AML) cells that led to the identification of a population of cells with a stem-like phenotype [15]. The authors identified a population of CD34^+^CD38^−^ AML stem cells that were capable of initiating AML in severe combined immune-deficient (SCID) mice. Although using a different set of markers, populations of CSCs were also found in other types of cancerous tissues, which confirmed their existence within other types of tumours and the role that they play in cancer initiation and progression [16,17,18,19,20,21,22,23,24,25,26,27,28,29,30,31,32,33,34,35]. Two main hypotheses have been proposed to explain how CSCs are originated within a tissue: the stochastic and the hierarchical models [36,37]. The stochastic model supports the hypothesis that any cell subjected to a specific set of somatic mutations could lead to the acquisition of self-renewal and differentiation properties that could contribute to tumour heterogeneity. On the other hand, the hierarchical model proposes that somatic mutations target specifically stem and progenitor cells that are hierarchically organized, resulting in the generation of self-renewing cancer stem and progenitor cells that produce differentiated progenies and recreate heterogenous tumours [38]. In summary, CSCs are populations of cancer cells that have the stem cell properties of self-renewal and ability to generate differentiated progenies.

## 3. Cancer Stemness, Therapy Resistance and Cancer Recurrence

**Figure 1:**
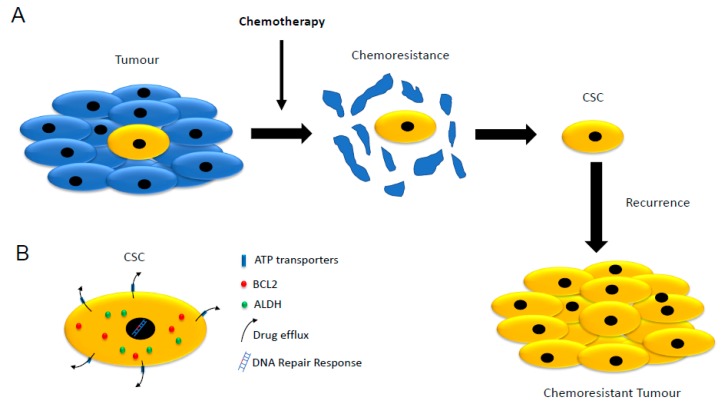
Schematic representation of cancer stem cells (CSCs) and their role in chemoresistance. (**A**) Chemotherapy destroys most of the highly proliferative cancer cells. CSCs are less affected by chemotherapy and can re-initiate cancer and promote cancer progression. (**B**) CSCs possess several mechanisms that counteract the effect of chemotherapeutic compounds such as the ATP family of transporters, higher expression of the pro-survival factor BCL2 (B-cell lymphoma 2), higher expression of the detoxifying enzymes aldehyde dehydrogenases (ALDHs), a more efficient DNA damage response and a slower rate of cell division or dormancy.

A major challenge in cancer therapy is associated with chemoresistance and recurrence following chemotherapy [2,39]. In most cases, this resistance is associated with the presence, within tumours, of aggressive cancer cell populations that developed mechanisms of chemoresistance responsible for unsuccessful therapies and leading to decreased survival rates of treated cancer patients (Figure 1A) [7,10]. Several cancer studies identified these aggressive cancer populations as cancer stem cells (CSCs) (Table 1) [15,16,40,41,42,43,44,45,46]. CSC-mediated chemoresistance relies on several cellular mechanisms that include lower proliferation rates and the ability to intracellularly and enzymatically inactivate chemotherapeutic compounds (Figure 1B) [7].

### 3.1. Low Proliferation Rate

Chemotherapy targets highly proliferative cells through DNA damage and inhibition of mitotic division; however, its action is limited when it applies to slow and non-dividing cancer cells such as CSCs [48,49,50,51]. Indeed, several studies have shown that CSCs, from different tissue origin, could resist the effect of a wide range of drugs such as doxorubicin, temozolomide, cisplatin, paclitaxel, etoposide and methotrexate [52]. Hence, following chemotherapy, which destroys the bulk of the tumour, CSCs remain on-site and can re-initiate tumorigenic and metastatic processes. Hence, developing therapies that target dormant or slow dividing cells is essential to prevent recurrent cancers.

### 3.2. Expression of ATP-Binding Cassette (ABC) Transporters

This large superfamily of membrane proteins functions in the transmembrane transport of various substrates by converting energy obtained from ATP hydrolysis [53]. Although the type of ABC transporters varies from one type of cancer to another, CSCs express high levels of these transporters, which contributes to an increased transport of chemotherapeutic compounds out of the cells, contributing therefore to CSC chemoresistance. Some ABC transporters are involved in the transport of a large number of drugs, while others transport only a few. For instance, the ABCC1 (ATP-binding cassette transporter C1) transporter also known as MRP1 (Multi Drug Resistance Protein 1), expressed by CSCs in glioblastoma, is involved in the efflux of a variety of therapeutic compounds including methotrexate, edatrexate, ZD1694, doxorubicin, daunorubicin, epirubicin, idarubicin, etoposide, vincristine, vinblastine, paclitaxel, irinotecan, SN-38, flutamide and hydroxyflutamide [54,55,56]. ABCB1 (ATP-binding cassette transporter B1), also known as MDR1 (Multidrug Resistance Protein 1), expressed by CSCs found in ovaries and breast cancers, acute myeloid leukaemia (AML), glioblastoma and renal cell carcinoma, provides drug multi-resistance to anthracyclines actinomycin D, colchicine, etoposide, teniposide, methotrexate, mitomycin C, mitoxantrone, paclitaxel, docetaxel, vincristine and vinblastine [57,58,59,60]. Other ABC transporters have a limited drug transport capacity, such as ABCA1 (ATP-binding cassette transporter A1), which is expressed by serous ovarian CSCs and which transports cisplatin [59]. In summary, ABCs are drug specific transporters that may require combination therapies that use inhibitors for the different transporters.

### 3.3. Increased Expression of Aldehyde Dehydrogenases (ALDHs)

This superfamily of enzymes is crucial for the detoxification of endogenous and exogenous aldehyde substrates by catalysing the oxidation of aldehydes to carboxylic acids [61,62,63]. They are highly expressed in CSCs from different types of cancers and their elevated levels of expression correlate with worse prognosis in cancer patients [64,65,66]. Although the mechanisms by which they promote CSC chemoresistance is not well known, this activity may involve their role in retinoic acid, reactive oxygen species and reactive aldehyde metabolisms [63]. The expression and activity of these enzymes have been used, together with other biomarkers, for the identification of CSCs in different types of cancers. For instance, breast cancer stem cells are identified using specific antibodies for CD44 (cluster of differentiation 44) and CD24 (cluster of differentiation 24), and an ADELFLUOR assay to detect ALDH activity. CD44^+^/CD24^−^/ALDH^+^ populations have been shown to possess characteristics of stem cells such as self-renewal, high proliferative potential, clonogenicity and multipotency [67,68,69,70]. In summary, ALDHs are associated with CSCs-mediated chemoresistance; however, their mechanisms of action are not well known. Thus, determining their modes of action will certainly help develop better targeting of these enzymes to prevent chemoresistance.

### 3.4. Resistance to Apoptosis

CCSs have been shown to resist apoptosis mediated by the intrinsic or mitochondria-dependent death pathway, and by the extrinsic cell death receptors pathway [71]. For instance, human glioma and leukaemia CSCs express lower levels of Fas and Fas ligand (Fas-L) resulting in resistance to extrinsic receptor-mediated cell death [72,73,74,75,76,77]. CSCs have also been shown to express higher levels of apoptotic inhibitors. The cellular Fas-associated death domain-like IL-1β-converting enzyme (FLICE)-inhibitory protein (c-FLIP) is highly expressed in leukaemia stem cells, and breast and glioblastoma CSCs [78,79,80]. C-FLIP averts apoptosis by preventing the formation of DISC (death-inducing signaling complex) and consequent activation of the caspase cascade via its interaction with FADD (Fas-associated death domain), caspase-8 or 10 and DR5 (Death receptor 5) [81,82]. Beside the extrinsic apoptotic pathway, the pro-survival protein Bcl-2 that is involved in the mitochondria-dependent death pathway, via its inhibitory interactions with the proapoptotic Bax (Bcl-2-associated x) and Bak (Bcl-2 antagonist killer), was found overexpressed in leukaemia, glioma and glioblastoma stem cells [83,84,85]. In summary, the inactivation of intrinsic and extrinsic cell death pathways ensures a selective survival advantage for CSCs. 

### 3.5. DNA Repair Response 

Another survival advantage in stressful conditions is related to CSCs’ capacity to timely activate the DNA damage sensor and repair machinery [86]. This process involves DNA repair pathways that include the nucleotide excision repair (NER), base excision repair (BER), mismatch repair (MMR), direct repair, and the double-strand break (DSB) recombinational repair. Evidence of CSCs’ resistance to radiotherapy was provided by a study that demonstrated the capacity of glioblastoma stem cells to efficiently activate the *ATM* serine/threonine kinase (ATM) and the DNA damage checkpoint protein kinase (Chk1) in response to ionising radiations [87]. Another study established the capacity of non-small cell lung cancer (NSCLC) stem cells to resist treatment with chemotherapeutic drugs by activating the Chk1 [88]. Mammosphere cells from the MCF-7 breast cancer cell line have been shown to have lower reactive oxygen species and a more active DNA single-strand break repair (SSBR) pathway, potentially associated to a higher level of expression of the key SSBR protein, human AP endonuclease 1 (Ape1) [89]. In conclusion, the enhanced DNA damage repair exhibited by CSCs appears to protect them from chemotherapy and radiotherapy-mediated apoptosis and adds another layer of protection to their survival capacities. Therefore, a better understanding of the mechanisms involved in DNA repair response will significantly contribute to improved therapy response and clinical outcome of patients treated with radiotherapy and DNA-targeting chemotherapies. 

## 4. CSC Microenvironment

**Figure 2:**
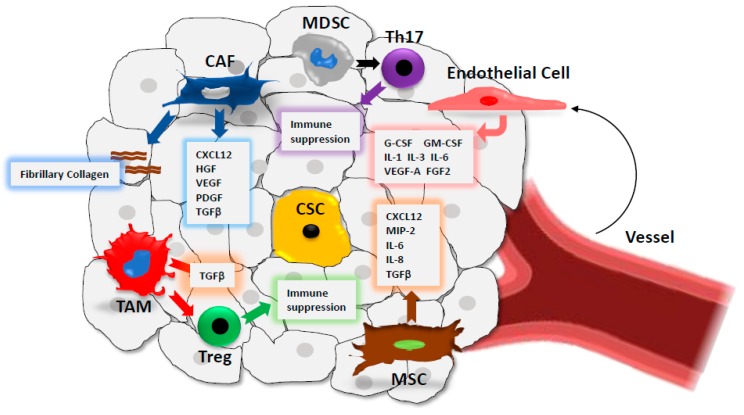
Schematic representation of the CSC microenvironment (CSC niche). The complex CSC niche contains several cell types including mesenchymal stem cells (MSCs), endothelial cells, cancer associated fibroblasts (CAFs) and immune cells (tumour associated macrophages (TAMs), regulatory T cells (Tregs), myeloid-derived suppressor cells (MDSCs) and T-helper cells (Th17)). These cells secrete different cytokines and growth factors that promote tumourigenesis, tumour progression and immunosuppression.

Stem cell niches represent areas of tissue that provide specific microenvironments, which maintain and promote CSCs’ capacity to self-renew and to generate differentiated progenies [90]. The concept of stem cell niche was first formulated by Schofield who demonstrated that successful transplants could only be successful if harvested from the bone marrow [91,92]. He also proposed that the stem cell niche is required for determining stem cell fate, as the behaviour of stem cells is influenced by their association with other cells within the niche. This concept was validated by other groups studying different tissues from invertebrate and vertebrate models [93,94]. This concept is applicable to cancer stem cells where the interaction with these niches is necessary for the maintenance of CSC populations. The CSC microenvironment is a highly heterogeneous complex comprised of cells such as stromal cells, immune cells and epithelial cells, and a network of extracellular macromolecules which provides support for the cells within the extracellular matrix (EC) (Figure 2) [95,96,97]. The cells found in the niche promote the growth, maintenance and differentiation of cancer stem cells. Currently, cancer therapies have been less successful as most of these drugs target the Bulk population of cancer cells, leaving cancer stem cell populations unaffected [98,99]. Therefore, understanding the relationship between CSCs and their microenvironment could help develop more efficient strategies to eliminate CSCs.

### 4.1. Cancer-Associated Fibroblasts (CAFs)

Although CAFs provide a mechanical supportive role for CSCs via enhanced production of fibrillary collagen, these cells also secrete the cytokine CXCL12 (C-X-C motif chemokine ligand 12) and growth factors such as the hepatocyte growth factor (HGF), the vascular endothelial growth factor (VEGF) and the platelet-derived growth factor (PDGF), which significantly contribute to CSCs’ increased proliferation, invasion and metastasis [97,100,101]. CAFs are also involved in cellular heterogenicity through secreted TGFβ1 (transforming growth factor beta 1), which promotes CSC related epithelial-mesenchymal transition (EMT), an early step of the invasive and metastatic process [102].

### 4.2. Immune Cells

Several immune cells contribute to the chronic inflammatory status of the CSC microenvironment, which enhances tumour proliferation, invasion and metastasis [103]. For instance, tumour-associated macrophages (TAMs) and myeloid-derived suppressor cells (MDSCs) secrete transforming growth factor beta (TGF-β), which contributes to EMT, invasion and metastasis [104,105,106,107]. In addition, immune cells within the microenvironment enhance tumour evasion via a variety of mechanisms. TAMs, via secreted TGFβ, recruit Tregs within the niche and contribute to immunosuppression. In a similar fashion, MDSCs, which secrete growth factors such as TGFβ, and cytokines recruit T helper 17 cells to promote their immunosuppressive activity [108]. Although we provide a very brief description here of the role of immune cells within the tumour microenvironment, a deeper discussion of their roles in this context is beyond the scope of the review.

### 4.3. Mesenchymal Stem Cells (MSCs)

MSCs are multipotent stromal cells that can differentiate into various cell types such as osteocytes, adipocytes and chondrocytes. These cells can migrate to chronic inflammatory sites such as cancer, where they contribute to metastasis by secreting TGFβ which promotes EMT [109]. An involvement in the colonisation process of secondary cancer sites has also been reported for metastatic breast, prostate and lung cancers [110,111,112,113]. MSCs have been shown to promote gastric cancer proliferation and angiogenesis via the secretion of VEGF, macrophage inflammatory protein-2 (MIP-2), TGF-β1, and the pro-inflammatory cytokines interleukin IL-6 and IL-8 [114]. Finally, MSCs may originate CAFs, which further contribute to CSC microenvironment cell heterogenicity and its metastatic potential [115,116,117].

### 4.4. Endothelial Cells

Angiogenesis is key for the CSC microenvironment as it delivers nutrients for CSC metabolism that are necessary for their self-renewal, and invasive and metastatic abilities. Through tumour vessels, immune cells such as T regulatory cells (Tregs) are recruited in situ and contribute to immune suppression [118]. Endothelial cells, together with perivascular cells, are the building blocks of vessels, and are stimulated by angiogenic factors such as the vascular endothelial growth factor (VEGF) and within a hypoxic environment such as cancer. This results in increased tumour vasculature and enhanced tumour growth and metastasis [119]. Additionally, tumour endothelial cells have been shown to secrete cytokines such as IL-3, granulocyte-CSF, granulocyte-macrophage-CSF (GM-CSF), IL-1, IL-6, VEGF-A, and bFGF, which promote and maintain CSC self-renewal and CSC-mediated progression [120,121].

## 5. Cancer Stemness, EMT and Tumour Progression

The main interest in investigating CSCs is related to their capacity to resist chemotherapy and radiotherapy, which results in cancer recurrence that is extremely difficult to overcome using currently available therapies. CSCs can re-initiate the tumorigenic process by generating a new tumour via their self-renewal and generation of differentiated cancer progenies. Another important property is their high invasive and metastatic potential, which involves a cellular process named EMT. Epithelial cells are connected to each other via lateral cell-cell junctions that include desmosomes, adherens junctions, tight junctions and gap junctions. During EMT, epithelial cells begin to trans-differentiate and start losing their lateral junctions and express molecules associated with the mesenchymal phenotype, resulting in the generation of mesenchymal cells [122]. These later play an essential role in the invasion process of adjacent tissues and distant organs via metastasis. The cytokine TGFβ, which is secreted in the CSC microenvironment by MSCs, CAFs and immune cells such as TAMs and MDSCs, plays an essential role in EMT-mediated CSC invasion [123]. TGF-β binding to its receptors results in the activation of the SMAD (small and mothers against decapentaplegic) proteins that translocate to the nucleus and activate target genes involved in EMT such as *SNAIL*, *bHLH* and *ZEB* factors [124,125]. These pro-EMT factors promote the expression of mesenchymal proteins such as N-cadherin, fibronectin and metalloproteinases (MMPs) [123]. Beside the TGFβ signalling, other signalling pathways, such as the WNT (wingless-related integration site), Notch (neurogenic locus notch homolog) and Hedgehog (HH) pathways, also participate in EMT. WNT signalling promotes EMT by stabilising β-catenin through the inhibition of glycogen synthase kinase-3β (GSK3β), resulting in β-catenin translocation to the nucleus and activation of the transcription factors lymphoid enhancer-binding factor 1 (LEF) and T cell factor (TCF), thus promoting a gene expression programme that favours EMT [126]. SHH signalling increases SNAIL1 expression in epithelial cancers leading to increased EMT and invasion [127]. Notch signalling may promote EMT through the Notch intracellular domain that directly activates SNAIL2 expression [128]. Finally, a crosstalk between TGFβ, WNT, Notch, Hedgehog (HH) and other signalling pathways such as RTK (receptor tyrosine kinase) signalling pathways may take place to promote EMT and invasion within the tumour microenvironment [129,130,131]. In summary, several pathways are involved in regulating EMT, and their targeting may require combination therapies that are directed against different components of this complex network, and with limited toxic effects.

## 6. CSC Metabolism

Cells require energy to perform their own processes of growth, division and survival. This need is met by the glycolysis metabolic pathway that converts glucose into pyruvate, two energy molecules of ATP and one of NADH (nicotinamide adenine dinucleotide hydrogen) [132]. The pyruvate is later transported in the mitochondria where it is converted into Acetyl-coA. The coenzyme produces the energy molecules NADH and FADH2 (flavin adenine dinucleotide hydrogen 2) through the Krebs cycle, which is also called the tricarboxylic acid cycle. The hydrogen ions that are used for the electron transport chain via the process of oxidative phosphorylation (OXPHOS) generate 36 molecules of ATPs. CSCs have been shown to have higher glycolytic metabolism when compared to normal and cancer cells. This difference could be due to higher expression of glycolytic enzymes such as GLUT1 (glucose transporter 1), HK-1 (Hexokinase-1) and PDK-1 (Pyruvate Dehydrogenase Kinase 1), and a higher consumption of glucose [132,133,134]. However, several studies challenged the concept of CSCs’ preferential reliance on the glycolytic metabolism pathway for energy production. These studies suggested that OXPHOS is more likely to be the main source of ATP due to increased mitochondrial ROS (reactive oxygen species), higher rates of oxygen consumption, and overall increase in mitochondrial function [135,136,137,138,139,140,141]. Overall, these opposing results could be explained by heterogenous differences in tissue origins, metabolic phenotypes and CSC adaptivity to environmental changes [142]. Therefore, until there is a better understanding of CSC metabolisms, developing targeted therapies remains a future therapeutic possibility.

## 7. Targeting Strategies of CSCs

CSCs are identified and isolated using cell markers whose expression create tissue-specific signatures. While some markers are commonly expressed by different types of cancer, others are tissue specific (Table 1). In this part, we provide an overview of the different approaches that were clinically employed to target CSCs and the results, so far, of past and ongoing clinical trials (Table 2).

### 7.1. Targeting Cell Surface Biomarkers of CSCs

#### 7.1.1. CD133

CD133 (cluster of differentiation 133 or human prominin-1), a marker that was first identified on the cell surface of CD34^+^ hematopoietic stem cells [143], has also been used to isolate populations of CSCs from glioblastoma, prostate, pancreas, ovarian, colon, lung and liver cancer (Table 1). The therapeutic targeting potential of CD133 was evaluated for advanced cholangiosarcoma, in a phase 2 clinical trial (NCT02541370) (CCA), and using an immunotherapeutic approach based on CAR-T cells (chimeric antigen receptor T cells) that were transduced with the anti-CD133 (CD133 CAR-T) and anti-EGFR (epidermal growth factor receptor) (CART-EGFR). Although the authors suggested that CART cocktail immunotherapy (CD133 CART and CART-EGFR) may be feasible for the treatment of CCA and other solid malignancies, resolving limitations that are associated with toxicities would need further investigation (Table 2) [144]. 

#### 7.1.2. CD44

CD44 is a cell surface adhesion receptor that is expressed by several types of cancers. The function of CD44 in growth and migration is dependent on its interaction with different ligands such as hyaluronic acid (HA), osteopontin (OPN), collagens and metalloproteases (MMPs) [145]. Targeting strategies for CD44 have led to the development of an antibody-based therapy that was clinically tested in a phase I study and on patients with refractory AML (acute myeloic leukeamia). The therapy using the humanised monoclonal antibody RG7356 resulted in limited clinical activity; however, the study validates the safety of the clinical use of the drug and suggests testing a combination therapy with other available drugs such as cytarabine (Table 2) [146].

#### 7.1.3. EpCAM

The epithelial cell adhesion molecule (EpCAM) is a transmembrane glycoprotein that is expressed by most human carcinomas [147]. The first EpCAM monoclonal antibody (edrecolomab) was tested in patients with metastatic colorectal cancer or in adjuvant settings and had a limited response in a defined number of patients with metastatic disease [148,149]. Unfortunately, subsequent larger studies could not confirm edrecolomab’s clinical activity in the adjuvant setting [150,151]. Other antibodies that were developed such as 3622W94 and ING-1 were discontinued in clinical trials due to poor tolerability (Table 2) [152]. Adecatumumab, a recombinant human IgG1 monoclonal, that was clinically tested in patients with metastatic breast cancer, resulted in a more favourable response in patients with higher expression of EpCAM compared to patients who had lower expression on their cell surface (Table 2) [153]. 

#### 7.1.4. CD47

The integrin associated protein (IAP) or CD47 (cluster of differentiation 47) was first identified as a β3 integrin interacting partner and was later found to interact with trombospondin-1 (TSP-1) and signal regulatory protein-alpha (SIRPα). Through these interactions, CD47 is involved in the regulation of various cellular functions such as cell migration, axon extension, cytokine production and T cell activation [154,155,156,157,158]. CD47 is overexpressed in most cancer types and was shown to promote cancer cell invasion and metastasis [159,160,161,162,163,164,165,166,167]. Two monoclonal antibodies have been developed to directly target CD47 in different types of cancer and were clinically tested as monotherapy or in combination with other monoclonal antibodies such as cetuximab and rituximab (Table 2) [168,169,170,171]. The first humanised anti-CD47 monoclonal antibody, Hu5F9-G4, was administered to patients with various solid tumours in a phase I clinical trial (NCT02216409). The trial aimed at optimising the dosage, the pharmacodynamic and the pharmacokinetic of the administered antibody. Although promising, the results were not statistically significant, and another clinical trial was performed with results not yet available (NCT02678338). The other monoclonal anti-CD47 monoclonal antibody CC-90002 was administered to patients with leukemia and myeloid and acute myelodysplastic syndromes in a phase I clinical trial, and the results are not yet published (NCT02641002). Both Hu5F9-G4 and CC-90002 are being clinically tested in combination therapies with the anti-EGFR antibody (cetuximab) or the anti-CD20 (cluster of differentiation 20) monoclonal antibody for the treatment of solid and haematological malignancies, and the trials are still ongoing (NCT02953782, NCT02953509, NCT02367196) (Table 2). 

### 7.2. Dendritic Cell-Based Vaccines

Dendritic cells (DCs) are antigen presenting cells that initiate and maintain immune responses through their essential function in anti-cancer immunity [172]. DCs present cancer antigens to effector T-cells, which leads to the targeting and eradication of cancer cells. They can also stimulate immunological memories that control cancer recurrence. These properties favour use in the development of dendritic cell-based immunotherapies [172,173]. Several dendritic cell-based vaccines have been tested in patients with glioblastoma multiforme in phase I and II clinical trials (Table 2). A phase I clinical trial study of a dendritic cell vaccine for newly diagnosed or recurrent glioblastoma patients resulted in adverse events and treatment-related toxicities (NCT02010606). This study used patients own immune-stimulating dendritic cells, which were isolated and treated to promote an immune response against cancer stem cells and injected under the skin in a series of vaccinations. Another phase I study investigated the use of CD133 peptides for a dendritic cell-based vaccine (ICT-121 DC vaccine), which was clinically tested in patients with glioblastoma multiforme (NCT02049489). In this study CD133 peptides were used to load DCs that were injected in patients to elicit an immune response. So far, the results of this study have not been published. An ongoing phase II study investigates the efficacy of autologous dendritic cells (DCs) that were loaded with autogeneic glioma stem-like cells (A2B5^+^) and were administered as a vaccination in adults with glioblastoma multiforme (primary or secondary) (NCT01567202). Finally, EPCAM-targeted CAR-T cells have been generated using genetically modified T cells that express a chimeric antigen receptor (CAR), and that bind to tumour cells expressing the EPCAM protein on the cell surface [174]. These CAR-Ts are being clinically tested in patients and for different types of solid tumours (NCT02729493, NCT02725125, NCT02915445 and NCT03013712).

## 8. Conclusions

Although progress has been made in developing cancer therapies that target CSCs, the specificity of the targeted antigens remains a challenge. Most of the therapies are directed against biomarkers that are not specifically expressed by CSCs and that are also found in normal cells within the body. This has unfortunate effects on increasing toxicity in treated patients. For instance, therapies that target CD133 resulted in adverse events and treatment-related toxicities in glioblastoma patients who were treated with a CD133-specific dendritic vaccine (NCT02010606). However, other ongoing therapies such as the ones combining two monoclonal antibodies or using EPCAM as a target may lead to better therapies. Making the data of the clinical trials available to the scientific and clinical communities will certainly help in sharing information for the development of more efficient targeting therapies (NCT02678338, NCT02641002, NCT02729493, NCT02725125, NCT02915445 and NCT03013712). Although cancer bi-therapies are certainly a better choice, further studies are required to identify more specific targets with a much lower toxicity when tested in clinical trials. Large scale analysis of tumour samples from different patients and cohorts using genomics, proteomics and data mining approaches (Bioinformatics) could lead to the identification of CSC-specific genes or antigen signatures that will allow the development of more efficient CSC targeted therapies with lower associated toxicities for treated patients. Furthermore, these approaches could also provide patient specific signatures that will allow a personalised therapy and reduced cost and toxicity.

Another aspect that has not been discussed in this review, is the role of epigenetics in regulating the role of CSCs in cancer initiation and progression. Non-coding RNAs such as microRNAs (miRNAs) and long non-coding RNAs (lncRNAs) function by regulating the expression of several CSC-specific genes. Other epigenetic regulators involve histone acetyl transferases (HATs) and histone deacetylases, which also play important roles in maintaining CSC stemness and differentiation [175,176]. Therefore, developing therapies, such as gene targeting, nanoparticles-based therapies, and HDAC inhibitors could also constitute a potential way forward for targeting CSCs.

In summary, whether using drugs, immunotherapies or gene therapies, the specificity of the targets is key for a successful eradication of CSCs and cancer recurrence.

## Figures and Tables

**Table 1:** Cell surface markers (solid tumours) that are used for the identification and isolation of CSCs.

Tumour	Biomarkers	References
Breast cancer	CD44^+^/CD24^−/low^/ALDH^+^	[16]
Prostate cancer	CD44^+^/a2b1^+^/ALDH^+^	[34]
Melanoma	ABCB5^+^	[17]
	CD20^+^	[18]
	CD271^+^	
Glioblastoma	CD133^+^	[20]
Colon cancer	CD133^+^/CD44^+^/ALDH^+^	[21]
	EpCAM^+^/CD44^+^/CD166^+^	[22]
	CD44^+^/CD24^+^	[23]
	CD133^+^/CD24^+^	[24]
Lung cancer	CD133^+^	
	CD44^+^	
	ALDH^+^	[35,47]
	CD117^+^	
Gastric cancer	CD133^+^	
	CD44^+^/CD24^+^	[25]
	CD90^+^	[26]
	CD44^+^/CD54^+^	
Head and neck cancer	CD44^+^/ALDH^+^	[27]
	CD44^+^/CD66^-^	
Ovarian cancer	CD133^+^	[28]
	CD44^+^	
	ALDH^+^	
	CD117^+^	
Pancreatic cancer	CD133^+^/CD44^+^/CD24^+^/ESA^+^	[29]
Liver cancer	CD133^+^/CD44^+^	[30]
	CD90^+^	[31]
	EpCAM^+^	[32]
	CD13^+^	[33]

**Table 2:** Clinically tested strategies that were employed to target CSCs. Two main therapies were used: monoclonal antibodies therapies and dendritic cells-based vaccines.

Target	Tumour Type	Therapy	Clinical Trial/Reports	Results
CD133	Advanced cholangiosarcoma	Cocktail CD133 CAR-T and CART-EGFR	NCT02541370 (Phase I and II)	Toxicity
	Glioblastoma multiforme	ICT-121 DC vaccine	NCT02049489 (Phase I)	Not published
CD44	Refractory Acute Myeloid Leukemia	Monoclonal antibody (RG7356)	146	Limited clinical activity
EpCAM	Metastatic colorectal cancer	Edrecolomab (Monoclonal antibody)	151	Limited response
			150	Not statistically significant
		3622W94 (Monoclonal antibody)	152	Toxicity
		ING-1 (Monoclonal antibody)		Toxicity
		Adecatumumab (Monoclonal antibody)	153	Favourable response in patients with highest expression
	Various types of tumours	EPCAM-targeted CAR-T cells	NCT02729493 (N/A)	Ongoing
			NCT02725125 (N/A)	Ongoing
			NCT02915445 (Phase I)	Ongoing
			NCT03013712 (Phase I and II)	Ongoing
CD47	Various solid tumours and hematological malignancies	Hu5F9-G4 (Monoclonal antibody)	NCT02216409 (Phase I)	Not statistically significant
			NCT02678338 (Phase I)	Not published
		Combination Hu5F9-G4 and Cetuximab	NCT02953782 (Phase I and II)	Ongoing
		Combination Hu5F9-G4 and Retuximab	NCT02953509 (Phase I and II)	Ongoing
		CC-90002 (Monoclonal antibody)	NCT02641002 (Phase I)	Ongoing
		Combination CC-90002 and Retuximab	NCT02367196 (Phase I)	Ongoing
Autogenic glioma stem-like cells (A2B5+)	Glioblastoma multiforme	Dendritic cell-based vaccine	NCT01567202 (Phase II)	Ongoing
